# Grants Recommended by the Convalescent Homes Committee

**Published:** 1918-12-21

**Authors:** 


**KING EDWARD'S HOSPITAL FUND—*(continued)*.**

### Grants Recommended by the Convalescent Homes Committee.

A.—CONSUMPTION SANATORIA.

*The Convalescent Homes Committee desire to draw attention to the fact that it must not be assumed that the reduction or absence of a grant implies dissatisfaction. (See paragraph 3 of Report.)*

CHILDREN'S SANATORIUM, Holt, Norfolk, £750, of which £700 in consideration of the reservation of ten children's beds during 1919 for the use of certain London hospitals (see paragraphs 5 and 6 of Report). DANESWOOD SANATORIUM (JEWISH), Woburn Sands, £450, of which £400 in consideration of the reservation of four beds during 1919 for the use of certain London hospitals (see paragraphs 5 and 6 of Report). Devon and Cornwall Sanatorium, Didworthy, South Brent, £1,150, of which £1,000 in consideration of the reservation of ten beds during 1919 for the use of certain London hospitals (see paragraphs 5 and 6 of Report). EVERSFIELD CHEST HOSPITAL, St. Leonards, £50. FAIRLIGHT SANATORIUM, Hastings, £50. KELLING SANATORIUM, Holt, Norfolk, £650, of which £600 in consideration of the reservation of six beds during 1919 for the use of certain London hospitals (see paragraphs 5 and 6 of Report). MOUNT VERNON HOSPITAL, Northwood, £150. NATIONAL ASSOCIATION FOR THE ESTABLISHMENT AND MAINTENANCE OF SANATORIA FOR WORKERS, Benenden, Kent, £3,250, of which £850 in reduction of mortgage debt, and £2,200 in consideration of the reservation of twenty-two beds during 1919 for the use of certain London hospitals (see paragraphs 5 and 6 of Report). Northamptonshire Sanatorium, Creaton, Northants, £1,325, of which £1,200 in consideration of the reservation of twelve beds during 1919 for the use of certain London hospitals (see paragraphs 5 and 6 of Report).

**Total for Consumption Sanatoria, £7,825.**

B.—CONVALESCENT HOMES.

*The names of Convalescent Homes receiving no grant are not published.*

BEAU SITE CONVALESCENT HOME, Hastings, £50. Brentwood Convalescent Home for London Children, Brentwood, £50. Brooklands Homes (Invalid Children's Aid Association), Worthing, £50. Bushey and Bushey Heath Children's Convalescent Home, Bushey, £50. CHELSEA HOSPITAL FOR WOMEN CONVALESCENT HOME, St. Leonards, £75, with a view to encouraging the admission without payment of patients direct from the hospital. Children's Convalescent Home, Beaconsfield, £25. Children's Cottage Hospital, Cold Ash, Newbury, £25, in consideration of convalescent cases. Children's Home Hospital, Barnet, £25, in consideration of convalescent cases. Convalescent Home for Poor Children, St. Leonards, £125. Convalescent Police Seaside Home, Hove, £50. E. G. ANDERSON HOSPITAL (HOME OF RECOVERY), Barnet, £150. FRIENDLY SOCIETIES' CONVALESCENT HOMES, Dover and Herne Bay, £50. GREAT NORTHERN CENTRAL HOSPITAL CONVALESCENT HOME, Hornsey, £25. HIGHGATE CONVALESCENT HOME FOR CHILDREN, Highgate, £50. Home for Convalescent Children, Southsea, £50. JEWISH CONVALESCENT HOME (CHILDREN), Brighton, £50. KING'S COLLEGE HOSPITAL CONVALESCENT HOME, Hemel Hempstead, £125. METROPOLITAN CONVALESCENT INSTITUTION, Bexhill, £75. Metropolitan Convalescent Institution, Walton, £150. Miller Hospital Convalescent Home, Bexhill, £50. NATIONAL HOSPITAL FOR THE PARALYSED AND EPILEPTIC CONVALESCENT HOME, East Finchley, £100, with a view to facilitating the admission of patients without payment. PADDINGTON GREEN CHILDREN'S HOSPITAL CONVALESCENT HOME, Slough, £100. Poplar Hospital Convalescent Home, Walton-on-Naze, £50. QUEEN CHARLOTTE'S LYING-IN HOSPITAL CONVALESCENT HOME, Kilburn, £50. Queen's Hospital for Children Convalescent Home, Bexhill, £150, of which £50 to reduce debt. ROYAL WATERLOO HOSPITAL FOR CHILDREN AND WOMEN CONVALESCENT HOME, Whippingham, £50. ST. ANDREW'S CONVALESCENT HOME, Folkestone, £50. St. Andrew's Convalescent Hospital, Clewer, £25. St. Luke's Home (Children), Woodley, Reading, £25, in consideration of convalescent cases. St. Mary's Convalescent Home, Birchington, £50. St. Mary's Home for Children, Broadstairs, £75, in consideration of convalescent cases. Samaritan Free Hospital Convalescent Home, Amersham, £25. Sunday School Union Convalescent Home, Bournemouth, £25. Surgical Home for Boys, Banstead, £25, in consideration of convalescent cases. TILFORD CONVALESCENT HOME FOR CHILDREN, Farnham, £50. VICTORIA HOME FOR INVALID CHILDREN, Margate, £25, in consideration of convalescent cases.

**Total to Convalescent Homes, £2,175.**

| | |
|---|---|
| **Grants to Consumption Sanatoria** | ... **£7,825** |
| **Grants to Convalescent Homes ...** | ... **£2,175** |
| **Total Grants for the Year 1918** | ...**£10,000** |

